# New Bioactive Peptides from the Mediterranean Seagrass *Posidonia oceanica* (L.) Delile and Their Impact on Antimicrobial Activity and Apoptosis of Human Cancer Cells

**DOI:** 10.3390/ijms24065650

**Published:** 2023-03-15

**Authors:** Diletta Punginelli, Valentina Catania, Giulia Abruscato, Claudio Luparello, Mirella Vazzana, Manuela Mauro, Vincenzo Cunsolo, Rosaria Saletti, Antonella Di Francesco, Vincenzo Arizza, Domenico Schillaci

**Affiliations:** 1 Section of Pharmaceutical Chemistry, Department of Biological, Chemical and Pharmaceutical Sciences and Technologies (STEBICEF), University of Palermo, Via Archirafi 32, 90123 Palermo, Italy; 2 Department of Earth and Sea Science (DiSTeM), University of Palermo, Viale delle Scienze Blg. 16, 90128 Palermo, Italy; 3 Section of Cell Biology, Department of Biological, Chemical and Pharmaceutical Sciences and Technologies (STEBICEF), University of Palermo, Viale delle Scienze Blg. 16, 90128 Palermo, Italy; 4 Section of Animal Biology and Anthropology, Department of Biological, Chemical and Pharmaceutical Sciences and Technologies (STEBICEF), University of Palermo, Via Archirafi 18, 90123 Palermo, Italy; 5 Department of Chemical Sciences, University of Catania, Viale A. Doria 6, 95125 Catania, Italy

**Keywords:** antibiotic resistance, drug-resistant bacteria, antimicrobial peptides, anticancer peptides, marine seagrasses, computational peptide design

## Abstract

The demand for new molecules to counter bacterial resistance to antibiotics and tumor cell resistance is increasingly pressing. The Mediterranean seagrass *Posidonia oceanica* is considered a promising source of new bioactive molecules. Polypeptide-enriched fractions of rhizomes and green leaves of the seagrass were tested against Gram-positive (e.g., *Staphylococcus aureus*, *Enterococcus faecalis*) and Gram-negative bacteria (e.g., *Pseudomonas aeruginosa*, *Escherichia coli*), as well as towards the yeast *Candida albicans*. The aforementioned extracts showed indicative MIC values, ranging from 1.61 μg/mL to 7.5 μg/mL, against the selected pathogens. Peptide fractions were further analyzed through a high-resolution mass spectrometry and database search, which identified nine novel peptides. Some discovered peptides and their derivatives were chemically synthesized and tested in vitro. The assays identified two synthetic peptides, derived from green leaves and rhizomes of *P. oceanica*, which revealed interesting antibiofilm activity towards *S. aureus*, *E. coli,* and *P. aeruginosa* (BIC_50_ equal to 17.7 μg/mL and 70.7 μg/mL). In addition, the natural and derivative peptides were also tested for potential cytotoxic and apoptosis-promoting effects on HepG2 cells, derived from human hepatocellular carcinomas. One natural and two synthetic peptides were proven to be effective against the “in vitro” liver cancer cell model. These novel peptides could be considered a good chemical platform for developing potential therapeutics.

## 1. Introduction

The dramatic increase in multidrug-resistant bacteria is becoming a serious threat for human health worldwide [1,2]. Recently, it has been estimated that antimicrobial resistance (AMR) caused 1.27 million deaths globally in 2019 [3], and major efforts to limit this problem are focused on the development of novel antibacterial agents which could elude microbial resistance by attacking new targets [4]. Apart from this serious problem, cancer also represents a global health concern worldwide: it has been reported that cancer caused 10 million deaths in 2020 [5], and this number is forecasted to exceed 13 million by 2030 [6]. The main limitation in the pharmacological treatment of various tumoral diseases is the resistance of cancer cells to conventional chemotherapeutic agents [7]. Hence, there is an urgent necessity to develop new drugs to overcome bacterial and cancer resistance, with high selectivity and an absence of adverse effects on normal cells [8,9]. 

Antimicrobial peptides (AMPs) are ancient effector molecules of the innate immune defense system of all living organisms, and they represent the first defense line against pathogens [10,11]. These small, amphipathic, cationic peptides are considered promising alternatives to overcome AMR due to their broad antimicrobial spectra, selective toxicity, and non-specific mechanism of action, which is based on targeting the main structural components of the bacterial membrane, thus reducing the development of microbial resistance to the bacteria compared to conventional antibiotics [12]. In addition, many AMPs have shown synergistic or adjuvant effects with common antibiotics [13,14], and they have been used as templates for in silico rational design of new, improved, and selective drugs [15]. In recent years, several studies have demonstrated that cationic AMPs can be considered a new class of antitumoral agents to counteract tumor resistance to conventional chemotherapy [16]. These molecules, also named anticancer peptides (ACPs), can be toxic to tumor cells, without any negative effects on healthy cells, through their ability to bind the phospholipid phosphatidylserines (PS) localized in the outer leaflet of plasma membrane in cancer cells [17]. 

To date, several AMPs and ACPs have been isolated from various natural sources such as insects and amphibians [7], although it is difficult to achieve representative quantities of peptides with the required purity for application in scientific and therapeutic research. Thus, the production of synthetic AMPs and ACPs represents a prominent strategy in the development of novel AMPs and ACPs with a wide range of bioactivities [18,19]. 

The marine environment and its biodiversity play a significant role as a source of new bioactive molecules, including novel AMPs and ACPs [20,21]. Among marine plants, seagrasses have recently received great attention due to the production of secondary metabolites that are considered of biomedical importance and could be used as potential pharmaceutical compounds [22,23].

The seagrass *Posidonia oceanica* (L.) Delile is an endemic plant in the Mediterranean Sea, and plays an important ecological role in marine ecosystem [24,25]. Few reported studies have illustrated the pharmacological properties of this seagrass, which is an important emergent depository of bioactive substances with antioxidant [26,27,28], antitumoral [29,30,31], antibacterial, and antifungal actions that are mostly related to their secondary metabolites (alkaloids, flavanoids, phenols, tannins, saponins, phlobatannins, sterols, proteins, reducing sugar polysaccharides and resins, etc.) [32,33,34]. In actuality, no studies as of yet have investigated the presence of AMPs in the leaves and rhizomes of *P. oceanica,* nor their role as biologically active compounds.

In the present study, we evaluated the antimicrobial and antibiofilm properties of polypeptide-enriched fractions derived from green leaves and rhizomes of *P. oceanica*. The extracts were screened for antimicrobial activity against Gram-positive and Gram-negative bacteria, as well as the yeast *C. albicans*. Nine novel peptides were identified in the extracts, and their amino acid sequences were characterized through nano-reversed phase ultra-high performance liquid chromatography (nanoRP-UHPLC), ultra-sensitive high-resolution mass spectrometry (HRMS), and a database search. Some natural and designed derivatives were tested for their antimicrobial and antibiofilm activities.

In addition, previous preliminary results have shown the cytotoxic and apoptosis-promoting effect exerted by extracts of the green leaves and rhizomes of *P. oceanica* on HepG2 cells. These cells are derived from human hepatocellular carcinoma, an aggressive cancer histotype constituting more than 90% of the cases of primary tumors of the liver (Abruscato et al., 2022 and manuscript submitted). Thus, the natural and derivative peptides were also tested for their potential anti-tumoral abilities.

## 2. Results

### 2.1. Antimicrobial and Antibiofilm Activity of P. oceanica Extracts

The antimicrobial activity of polypeptide-enriched extracts isolated from green leaves and rhizomes of *P. oceanica* was evaluated, starting with a 50% *v*/*v* concentration of each sample against four bacterial strains (*S. aureus* ATCC 25923, *P. aeruginosa* ATCC 15442, *E. coli* ATCC 25922, and *E. faecalis* ATCC 13813) and a yeast (*C. albicans* ATCC 10231). The results are expressed in terms of minimum inhibitory concentration (MIC), reporting the values in percentage (*v*/*v*) and in concentration (μg/mL) of protein content (Table 1).

The extract of the green leaves showed a MIC of 25% *v*/*v* (corresponding to a protein content of 7.5 μg/mL) against three selected strains, and a value of 12.5% *v*/*v*, equal to 3.75 μg/mL, against *P. aeruginosa* ATCC 15442. Different MIC values were detected for the rhizome extract, which showed a MIC of 12.5% *v*/*v* (corresponding to a protein content of 3.25 μg/mL) against three selected strains and a MIC of 13 μg/mL and 1.61 μg/mL towards *S. aureus* ATCC 25923 and *P. aeruginosa* ATCC 15442.

The inhibition of biofilm formation by both polypeptide-enriched extracts of the seagrass against *S. aureus* ATCC 25923, *P. aeruginosa* ATCC 15442, and *C. albicans* ATCC 10231 was assessed at sub-MIC concentrations, starting from 5% *v*/*v* and reporting the values in terms of Biofilm Inhibition Concentration 50% (BIC_50_), that is, the concentration at which an inhibition of 50% of the biofilm is encountered. The assays showed that the extract of the green leaves of *P. oceanica* inhibited biofilm formation of *S. aureus* ATCC 25923 and *C. albicans* ATCC 10231, with a BIC_50_ equal to 0.67 μg/mL and 0.94 μg/mL, respectively, whereas the extract was inactive against *P. aeruginosa* ATCC 15442. Moreover, the polypeptide-enriched extract from the rhizomes of *P. oceanica* revealed indicative inhibition of the biofilm formation in *S. aureus* ATCC 25923 and *C. albicans* ATCC 10231, with BIC_50_ values equal to 0.85 and 0.36 μg/mL, respectively, whereas no inhibition was detected for the biofilm formation of *P. aeruginosa* ATCC 15442 (Table 2). 

Both polypeptide-enriched extracts of *P. oceanica* were further analyzed through nRP-UPLC-High Resolution nESI MS/MS analysis in order to assess the presence of potential peptides. 

### 2.2. MS Analysis of the Peptide Sequences Identified in P. oceanica Extracts

The aforementioned extracts of *P. oceanica* were submitted to nRP-UPLC-High Resolution nESI MS/MS analysis with the goal of characterizing the amino acid sequence of the identified peptides. MS data were analyzed through PEAKS software packages, which performed a database search using the de novo sequencing strategy. Overall, nine novel peptide sequences were characterized in both polypeptide-enriched extracts of the seagrass; in particular, four sequences were identified in the green leaf sample, whereas five were found in the rhizome extract (Table 3). In addition, the APD3 “Antimicrobial Peptide Calculator and Predictor” tool was used to predict their potential ability to act as antimicrobial peptides, comparing sequences and similarities with already-studied AMPs [35]. By this approach, four out of nine peptides might be acting as potential AMPs due to their predicted ability to interact with the bacterial membrane. Two peptides were from the green leaf extract (i.e., peptides #2 and #3) and two from the rhizome sample (peptides #5 and #7). Therefore, these peptides were selected for further analyses to assess their physico-chemical parameters (Table 4) using the antimicrobial peptide database [35] and half-life of peptide, a significant tool that enables the prediction of the half-life of a peptide in a biological proteolytic environment [36]. The two peptides identified in green leaf extract of the seagrass showed a negative net charge and a good hydrophobic ratio (i.e., 38 and 43%, respectively) and Boman Index (≤2.5 kcal/mol) [37,38]. On the contrary, the two peptides (peptides #5 and #7) discovered in the rhizome extract were characterized by a neutral net charge, a higher hydrophobic ratio (i.e., 63 and 50%, respectively), and a different Boman Index. Moreover, both peptides derived from green leaf extract of *P. oceanica* display higher values of Wimley–White whole residues with respect to the peptides identified in the rhizome extract. High Wimley–White values indicate a better ability to interact with and perturb the bacterial membrane [35]. Other parameters of interest are illustrated in Table 4. 

### 2.3. AMP Prediction and Characterization 

Potential biological properties of selected peptides of *P. oceanica* were assessed through seven available prediction models: the servers DPABBs [39] and CellPPd [40,41] to detect the antibiofilm and cell-penetrating abilities of the aforementioned peptides; the Predicted Antigenic tool [42] to evaluate the potential presence of antigens; the server HemoPI [43] to forecast the hemolytic activity of a peptide; ToxinPred [44,45] to discriminate the toxic peptides; a peptide cutter server [46], an important tool to predict the presence of cleavage sites; and the server iAMPpred [47], to estimate the antimicrobial and antifungal activity. 

According to the predictions of the server models CellPPd and dPABBs, the selected sequences did not show any ability to penetrate bacterial cell and antibiofilm activity, except for peptide #2, which may have displayed antibiofilm properties. Other potential activities of the peptides are reported in Table 5.

### 2.4. Antimicrobial and Antibiofilm Activity of the Natural Peptides

In spite of server predictions, antimicrobial and antibiofilm assays demonstrated that the selected natural peptides (chemically synthesized) did not display any activity in vitro at the maximum tested concentration (MIC > 250 μg/mL) against *S. aureus* ATCC 25923, *P. aeruginosa* ATCC 15442, *E. coli* ATCC 25922, *E. faecalis* ATCC 29212, or *C. albicans* ATCC 10231. To exclude the potential interference of salt-mediated inactivation of AMPs related to conventional medium content, we evaluated the half-maximal effective concentration (EC_50_ concentration to inhibit 50% of viable bacteria) by testing the peptides under low-salt conditions in 10 mM sodium phosphate buffer. The selected peptides did not show any activity at the maximum tested concentration of 100 μg/mL (EC_50_ > 100 μg/mL).

**Figure 1:**
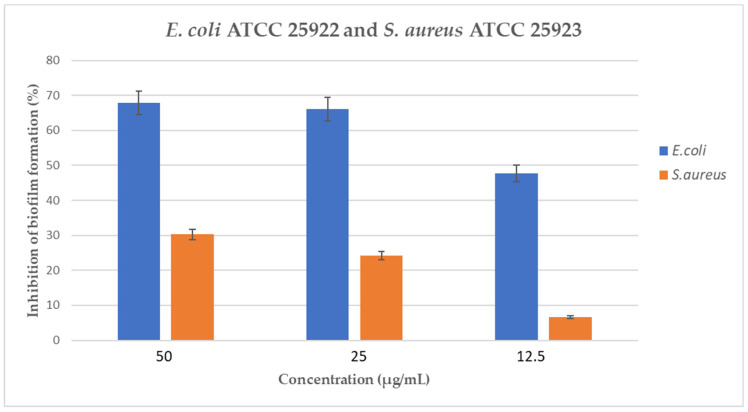
Inhibition of biofilm formation by peptide #3 against *E. coli* ATCC 25922 and *S. aureus* ATCC 25923.

Among the discovered peptides, only peptide #3 (NVVELNVAPGDK) showed an interesting inhibition of biofilm formation in *E. coli* ATCC 25922 and a weak one in *S. aureus* ATCC 25923, with percentages of inhibition equal to 67.83% and 30.3%, respectively, at the tested peptide concentration of 50 μg/mL (Figure 1). The value of BIC_50_ for *E. coli* ATCC 25922 was equal to 17.6 μg/mL. 

With the aim to improve the antimicrobial and antibiofilm properties of selected natural peptides of *P. oceanica*, further bioinformatic analyses were carried out.

### 2.5. Optimization of the Antimicrobial Properties of the Synthetic Peptides through Bioinformatic Analysis

To investigate how to improve the physico-chemical and biological parameters, as well as the antimicrobial activity of peptides #2, #3, #5, and #7, their amino acid sequences were modified through bioinformatic analysis. In particular, by substitution or deletion of some amino acidic residues, we designed four new synthetic derivative peptides characterized by a net positive charge ranging from +1 to +3; a hydrophobic ratio > 40%; and a Boman Index value, which assesses the protein-binding affinity domain, lower than 2.5 kcal/mol (Table 6). In addition, the potential biological features of selected synthetic derivative peptides were also analyzed through online webservers (Table 7). According to the predictions, two synthetic derivative peptides (peptide #5d and peptide #7d) revealed significant antimicrobial and antifungal activity (in percentage) compared to Peptide #2d, whereas Peptide #3d showed only indicative antimicrobial properties. Moreover, all synthetic derivative peptides displayed antibiofilm activity, low hemolytic and toxic potential, and presence of cleavage sites. 

### 2.6. Antimicrobial and Antibiofilm Activity of Synthetic Derivative Peptides of P. oceanica

**Figure 2:**
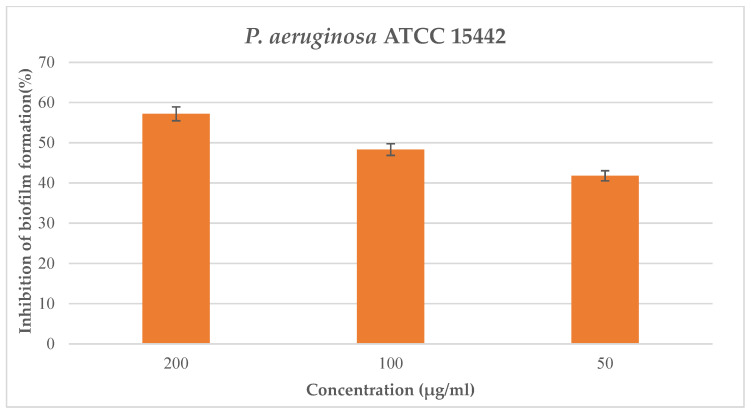
Inhibition of biofilm formation by synthetic derivative peptide #5d against *P. aeruginosa* ATCC 15442.

The synthetic derivative peptides #2d, #3d, #5d, and #7d were assessed in vitro against four bacterial strains (*S. aureus* ATCC 25923, *P. aeruginosa* ATCC 15442, *E. coli* ATCC 25922, and *E. faecalis* ATCC 29212) and one yeast strain (*C. albicans* ATCC 10231), but showed no antimicrobial or antifungal activity at the maximum concentration tested of 250 μg/mL. Moreover, the aforementioned peptides were evaluated for their ability to inhibit biofilm formation of the selected bacterial and yeast strains; none of these revealed antibiofilm properties against the cited pathogens at the maximum tested concentration of 200 μg/mL. In contrast, the synthetic derivative peptide #5d showed notable interference with biofilm formation of *P. aeruginosa* ATCC 15442 at the maximum tested concentration of 200 μg/mL, with a percentage of inhibition formation equal to 57.2% (Figure 2) and a value of BIC_50_ equal to 70.7 μg/mL.

### 2.7. Cytotoxic and Apoptosis-Promoting Effects of Peptides #7, #3d, and #7d on Tumor Cells

In a final set of experiments, we examined the effect of incubation with natural and synthetic peptides on HepG2 cell viability via trypan blue exclusion assay, evaluating the IC_50_ if cytotoxicity occurred. Among all the tested peptides, only one natural peptide and its synthetic derivative, i.e., #7 and #7d, as well as an additional synthetic peptide, i.e., #3d, determined a dose-dependent decrease in cell viability after 24 h of exposure, with an average IC_50_ of 329, 108, and 410 ng/mL, respectively.

**Figure 3:**
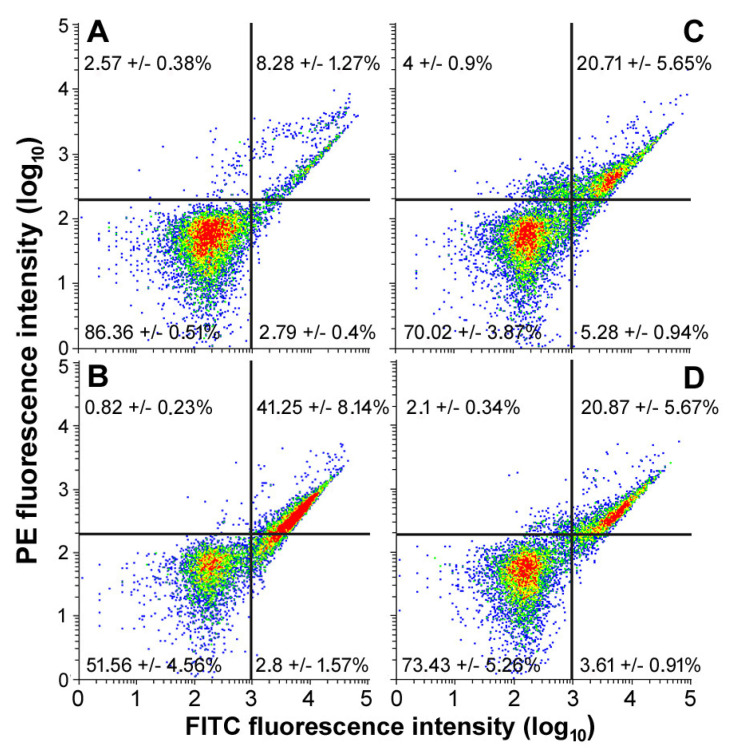
Flow cytometric assays for apoptosis in HepG2 cells cultured under control conditions (**A**) or exposed to peptide #7 (**B**), #3d (**C**), and #7d (**D**) at their respective IC_50_ values for 24 h. The plots show the results of representative experiments. The percentages indicated as the mean ± s.e.m. of three independent experiments refer to viable annexin-V^−^/PI^−^ cells (**bottom left quadrant**), early apoptotic annexin-V^+^/PI^−^ cells (**bottom right quadrant**), late apoptotic annexin-V^+^/PI^+^ cells (**top right quadrant**), and necrotic annexin-V^−^/PI^+^ cells (**top left quadrant**).

Cultures grown in control conditions or exposed to either peptide at their respective IC_50_ for 24 h were assayed for the externalization of phosphatidylserine using recombinant annexin-V conjugated to green fluorescent FITC dye in conjunction with propidium iodide (PI) in order to discriminate viable, early apoptotic, late apoptotic, or necrotic cells. The obtained data are shown in Figure 3 and indicate that peptide #7 and, to a lesser extent, peptides #7d and #3d are capable of triggering programmed cell death in HepG2 cells. In particular, the percentage of viable annexin-V^−^/PI^−^ cells decreased from about 86% of the controls to about 51% after treatment with the natural peptide, and to about 70–73% when exposed to both derivative peptides. On the other hand, the proportion of late apoptotic cells (annexin-V^+^/PI^+^) increased from 8% to about 41 (for peptide #7) and 20% (for both synthetic peptides) of the population. No substantial increase was found for the percentages of early apoptotic (i.e., annexin-V^+^/PI^−^) and necrotic (i.e., annexin-V^−^/PI^+^) cell populations under the experimental conditions used.

## 3. Discussion

There is an urgent need to discover and develop new therapeutics to combat antimicrobial resistance (AMR). We also need novel antimicrobials that can oppose multifactorial resistance to conventional antibiotics associated with biofilm formation and the growth of most pathogens. Natural AMPs are a good candidate for an alternative to conventional antibiotics or adjuvants of antibiotics in the treatment of multi-resistant bacterial and fungal infections, although they show some negative features, such as toxicity, low resistance to proteolysis, and a high cost of isolation and purification. The rational design of synthetic antimicrobial peptides (SAMPs) may be considered an alternative solution to overcome these disadvantages, since they show low or no toxicity to mammalian cells and are less prone to trigger antimicrobial resistance [48,49]. 

With the aim to detect novel molecules with antimicrobial properties, we focused in the past on polypeptide-enriched extracts from different organisms (e.g., invertebrates, plants, terrestrial fungi), identifying and characterizing several natural peptides with potential antimicrobial activity [50,51]. In this study, polypeptide-enriched extracts from the Mediterranean seagrass *P. oceanica* were evaluated against relevant bacterial and fungal pathogens, and nine peptides were identified by coupling nano-reversed phase ultra-high performance liquid chromatography (nanoRP-UHPLC) and ultra-sensitive high-resolution mass spectrometry (HRMS). Among them, peptides #2, #3, #5, and #7 showed a good similarity to host defense peptides identified in other organisms (e.g., bacteria, invertebrates, vertebrates); however, only peptide #3 demonstrated a prominent inhibition of biofilm formation against bacterial pathogenic strains *E. coli* ATCC 25922 and *S. aureus* ATCC 25923, with percentages of inhibition equal to 67.83% and 30.3%, respectively. 

It has been reported that natural peptides may possess some disappointing characteristics such as instability, immunogenicity, hemolytic activity, host toxicity. Therefore, in order to face the drawbacks of natural peptides, in this study, derivative peptides were designed, referring to natural peptides as templates. Through bioinformatic tools and online servers based on several algorithms, the physicochemical parameters of these natural peptides were modified by changing, deleting, or adding specific amino acids with the aim of improving antimicrobial properties, selectivity, and stability. However, the peptide derivatives #2d, #3d, #5d, and #7d showed disappointing antimicrobial activity in vitro compared to what was predicted by bioinformatic analysis. The abundance of generated sequences obtained through computational methods makes the choice of the best amino acidic sequence for in vitro biological tests difficult. However, the predicted low toxicity and stability in the host environment are the main criteria for sequence selection, promoting shorter peptides (<15 amino acids) that make chemical synthesis easier through reducing production costs [52].

Among the synthetic derivative peptides, we observed that only peptide #5d, selected on the basis of three main physicochemical parameters (percentage of hydrophobic ratio, net positive charge, and Boman index) showed interesting antibiofilm activity, and it may be considered a potential effective antimicrobial agent to counteract the pathogen *P. aeruginosa*.

It is known that hepatocellular carcinoma, estimated as the fifth most common type of cancer worldwide and the second leading cause of death due to cancer in men, displays a generally poor prognosis, as well as that resection and transplantation are still considered as cornerstone treatment options [53]. Thus, significant efforts are being directed toward the development of new drugs, mainly from natural sources, that are able to exert minimal adverse effects along with the maximal therapeutic activity. In this framework, it is acknowledged that AMPs may exhibit a wide range of biological activities and targets, including cancer cells, which are addressed to cytotoxicity and, frequently, apoptotic death, due to the peptides’ membranolytic ability exerted by their binding to external phosphatydilserine phospholipids [54]. The HepG2 cell line, obtained from a bioptic fragment of a young Caucasian male affected by differentiated hepatocellular carcinoma, represents one of the most widely studied “in vitro” model systems for bio-toxicological studies on this tumoral histotype [55]. In recent years, we have demonstrated the cytotoxic effect of different aqueous extracts from marine species on human cancer cells [56,57,58,59]. In particular, preliminary results have shown that water-soluble preparations from the leaves and rhizomes of *P. oceanica* cause HepG2 cells to undergo programmed cell death [60]. Herein, we tested the viability-restraining and apoptosis-promoting ability of a panel of natural and synthetic peptides from this seagrass and found three in particular, i.e., #7, #3d, and #7d, that can cause cell death in HepG2 cells via apoptosis induction. 

Limited information is available in the literature on plant-derived anticancer peptides that are active on HepG2 cells. The peptide EQRPR, able to impair their proliferation, was isolated from rice bran [61]. Preparations of corn peptides were found to exert growth-restraining, cell cycle-damaging, and apoptosis-promoting effects, and the “in vitro” results were corroborated by evidence of “in vivo” inhibition of hepatocellular carcinoma [62]. Apoptosis was also triggered by peptides isolated from mung beans and rapeseed protein hydrolysates [63,64]. To our knowledge, the results reported herein, which add themselves to this short list, represent the first evidence of small linear anti-cancer peptides, both natural and modified, derived from a marine plant species.

The content of hydrophobic amino acids in the peptides is essential to allow their interaction with the lipid bilayer of the cancer cell plasmalemma. Interestingly, Li et al. (2019) [64] reported that most anticancer peptides from food are predominantly composed of Gly, Leu, Ala, and Pro, secondarily containing one or more residues of Glu, Lys, Arg, and Thr. This selective requirement of favorite amino acids may account for the inability of peptide #5 to exert any biological role, despite being endowed with the highest hydrophobic ratio. On the other hand, the low hydrophobic ratio of peptide #2 was conceivably responsible for its lack of activity, whereas the increase in this ratio in peptide #7d vs. its natural counterpart might explain the gain of cytotoxic function by the synthetic derivative.

## 4. Materials and Methods

### 4.1. P. oceanica Collection and Extract Preparation

*P. oceanica*’s green leaves and rhizomes were harvested manually after a storm along the coast of Isola delle Femmine (Palermo, Italy) in April 2019. The samples were kept in a salt-water tank and transported to the laboratory, where they were accurately washed with fresh water to remove epiphytic components. Green leaves and rhizomes were crushed separately in a mortar with the gradual addition of liquid nitrogen, in order to obtain fine powders. Then, polypeptide fractions of these powders were extracted using acetic acid (2 M) with a 1:3 ratio, adding antiproteases (1:200). Lastly, the samples were homogenized, sonicated, and centrifuged in order to achieve the polypeptide fraction. 

### 4.2. Protein Concentration Assessment

The protein concentrations of green leaf- and rhizome-derived powdered samples were determined using the Bradford method [65], adding 900 mL of Bradford to 100 μL samples and evaluating the absorbance of the preparations at a wavelength of 595 nm through a spectrophotometer. 

### 4.3. Bacterial Strains 

Four bacterial strains and one fungal strain were employed in this study: *S. aureus* ATCC 25923, *P. aeruginosa* ATCC 15442, *E. coli* ATCC 25922, *E. faecalis* ATCC 29212, and *C. albicans* ATCC 10231. The media used for microbiological assays were Tryptic Soy Broth (TSB, Sigma-Aldrich, Merck-Life Sciences S.r.l., Milan, Italy), Tryptic Soy Agar (TSA), Mueller Hinton II (Sigma-Aldrich, Merck-Life Sciences S.r.l., Milan, Italy), and Sabouraud medium for the yeast strain.

### 4.4. Determination of Minimum Inhibitory Concentration (MICs) 

Polypeptide-enriched extracts derived from green leaves and rhizomes of *P. oceanica* were lyophilized and resuspended in ultrapure water in order to obtain protein concentrations of 30 μg/mL and 26 μg/mL, respectively. The MIC values of the extracts were assessed through a previously described micro-method [66] based on serial dilution (1:2) of the extracts, using Mueller Hinton II (MH II) and Sabouraud (for *C. albicans*) media in 96-well microplates and starting from 50% *v*/*v* to 1.5% *v*/*v* for a final volume equal to 100 μL. A microbial suspension of 10 μL (5 × 10^6^ CFU/mL) derived from a bacterial culture grown at 37 °C for 24 h on Tryptic Soy Agar (TSA) in 5 mL of NaCl 0.9%, whose turbidity was similar to the 0.5 McFarland standard, was added to a 96-well plate followed by incubation at 37 °C for 24 h. The MIC values of the extracts were assessed through a microplate reader (Glomax^®^-Multidetection System, Promega Italia, S.r.l., Milan, Italy), and the lowest concentration of the sample, whose optical density (OD) was 570 nm, was equivalent to the negative control wells (broth without bacterial inoculum to control the sterility of medium). In addition, a positive growth control (broth with bacterial inoculum to compare the growth of the cells with samples) and a sample control (extract solution without bacterial inoculum to verify the absorbance of the samples at various tested concentrations) were also included. 

The same procedure and media were also used to evaluate the antimicrobial activity of synthetic peptides #2, #3, #5, and #7, as well as of their derivatives. In particular, the synthetic peptides and their derivatives were dissolved in ultrapure water with the goal of achieving a stock solution of 5 mg/mL. Work solutions of 250 μg/mL of each synthetic peptides and derivatives were prepared in MH II and Sabouraud, being tested in 96-well microplates after serial dilution (1:2). Each assay was performed in triplicate [50].

### 4.5. Biofilm Inhibition Formation (Crystal Violet Method)

Sub-MIC concentrations of natural extracts and synthetic peptides were tested to evaluate their interference with the growth as a sessile community of *S. aureus* ATCC 25923, *P. aeruginosa* ATCC 15442 and *C. albicans* ATCC 10231, as previously reported [67]. Bacterial strains or fungal strains were incubated in test tubes containing TSB or Sabouraud (5 mL) and 2% *v*/*v* glucose at 37 °C for 24 h. Then, a 2.5 μL microbial suspension was added to each well, containing 200 μL of TSB or Sabouraud broth with 2% *w*/*v* glucose. [68,69]. Aliquots of sub-MIC concentrations of natural extracts of *P. oceanica* or of chemically synthesized peptides were directly added to the wells, and the plates were incubated at 37 °C for 24 h. After biofilm formation, the wells were washed twice with sterile NaCl 0.9% and stained with 200 μL of 0.1% *v*/*v* crystal violet solution for 30 min at 37 °C [70]. Excess solution was discharged, and the plates were washed twice using tap water. Lastly, each stained well was processed by adding 200 μL of ethanol to solubilize the dye. The OD was read at a wavelength of 600 nm using a microplate reader (Glomax^®^-Multi Detection System, Promega s.r.l., Milan, Italy). The experiments were carried out at least in triplicate, and three independent experiments were performed. The percentage of biofilm inhibition formation was determined through the following formula:

% Inhibition = [(OD growth − OD sample)/OD growth control] × 100.

BIC_50_, which represents the concentration at which the percentage of biofilm inhibition formation is equal to 50%, was calculated using an AAT Bioquest, Inc. Quest Graph^TM^ IC_50_ Calculator (v.1), retrieved from https://www.aatbio.com/tools/ic50-calculator-v1 (accessed on 1 June 2022) [71].

### 4.6. Mass Spectrometry Analysis 

Mass spectrometry investigations were performed using an Orbitrap Fusion Tribrid^®^ (Q-OT-qIT) mass spectrometer (Thermo Fisher Scientific, Bremen, Germany) coupled on-line with a Dionex UltiMate 3000 RSLCnano system. Particularly, nHPLC-nESI MS/MS analyses were carried out using 1 μL (corresponding to 25 ng) of each solution, as previously reported [50]. Precursor peptides were analyzed from 400 to 1600 *m*/*z* in high resolution mode (resolution of 120 K, @ 200 *m*/*z*). Only those precursors with charge states 1 ÷ 4 were sampled and fragmented for MS/MS analysis; this was obtained in low resolution mode using a linear trap analyzer [50].

### 4.7. Database Search 

MS/MS data were processed and searched using the PEAKS de novo sequencing software (v. 10.0, Bioinformatics Solutions Inc., Waterloo, ON, Canada). The database search was restricted to the *Viridiplantae* taxonomy (40656 entries, December 2020 release) of the reviewed section (Swiss-Prot) of UniProt sequence resource (https://www.uniprot.org/, URL accessed on 2 January 2022), and used the same parameters reported in [50]. Finally, all de novo sequences and the corresponding peptides–spectrum matches were manually checked.

### 4.8. AMP Prediction by Bioinformatic Analysis

The physico-chemical parameters of the natural sequences and synthetic peptides were determined through the webserver “APD3 Antimicrobial Peptide and Calculator” tool included in the antimicrobial peptide database (APD) [35]. The predicted half-life and stability of the aforementioned sequences in an intestine-like proteolytic environment were assessed using “HLP: Webserver for predicting half-lifes of peptides in intestine-like environment” [36]. The biological properties of the synthetic peptides were evaluated using online free servers: (1) dPABBs (https://ab-openlab.csir.res.in/abp/antibiofilm/, accessed on 1 March 2022) [39]; and (2) CellPPd (https://webs.iiitd.edu.in/raghava/cellppd/index.html, accessed on 1 March 2022) [40,41], which allowed the design of cell-penetrating peptides. The antibacterial and antifungal features were analyzed using the iAMPpred tool (http://cabgrid.res.in:8080/amppred/, accessed on 1 March 2022) [47], whereas the hemolytic and toxic properties were evaluated through HemoPI (https://webs.iiitd.edu.in/raghava/hemopi/, accessed on 1 March 2022) [43] and ToxinPred (https://webs.iiitd.edu.in/raghava/toxinpred/, accessed on 1 March 2022) [44,45], respectively. Additionally, the allergic potential was assessed using the antigenic prediction tool (http://imed.med.ucm.es/Tools/antigenic.pl, accessed on 1 March 2022) [42]. The isoelectric point (PI) (https://web.expasy.org/protparam/, accessed on 1 March 2022), the presence of cleavage sites (https://web.expasy.org/peptide_cutter/, accessed on 1 March 2022), and the molecular mass (https://aps.unmc.edu, accessed on 1 March 2022) were also assessed [38].

### 4.9. Peptide Synthesis

The tested peptides were synthesized by GenScript Biotech (Leiden, The Netherlands), basing on our indications. The peptides were gained through fluorenylmethyloxycarbonyl protecting group (Fmoc) solid phase technology. The quality and purity of the peptides (≈98%) were assessed through mass spectrometry (MS) analyses and high-performance liquid chromatography. The obtained powdered peptides were kept at −20 °C for storage.

### 4.10. HepG2 Cell Culture and IC_50_ Evaluation

HepG2 human hepatic cancer cells (taken from laboratory stocks) were routinely grown in high glucose–DMEM medium with the addition of 10% fetal calf serum (Life Technologies, Carlsbad, CA, USA) and antibiotics (100 U/mL penicillin and 100 μg/mL streptomycin; Life Technologies), at 37 °C in a 5% CO_2_ atmosphere.

For IC_50_ determination, cells undergoing exponential growth were seeded at a concentration of 5500/well in 96-well plates, allowed to adhere overnight, and then grown in control conditions or exposed to different concentrations of natural and synthetic peptides for 24 h. The number of viable cells was determined through the trypan blue exclusion test [59,72]. The cell viability ratio in each experimental condition was determined as the ratio between unstained treated cells and controls, and the half maximal inhibitory concentrations (IC_50_) for peptides #7, #3d, and #7d were evaluated using the CompuSyn software [73].

### 4.11. Annexin V-FITC Binding Assay

HepG2 cells were grown for 24 h in control conditions or exposed to peptides #7, #3d, and #7d at their respective IC_50_ values and the percentage of control, and treated cells undergoing apoptosis were evaluated with the Annexin V-FITC kit (Canvax Biotech, Cordoba, Spain), following the manufacturer’s instructions. Three independent experiments were performed, as described in [58], using a FACSCanto flow cytometer (BD Biosciences, Franklin Lakes, NJ, USA) and evaluating 10,000 events. The resulting .fcs files were analyzed with the online Floreada tool (https://floreada.io, accessed on 1 January 2023).

## 5. Conclusions

Two synthetic peptide derivatives showed interesting antibiofilm activity against two common bacterial pathogens (*P. aeruginosa* and *E. coli*). 

One natural and two synthetic peptides were proven to be effective against the “in vitro” liver cancer cell model. Thus, the collective results obtained herein open up new and diversified potential scenarios for the future biomedical applications of the discovered and designed peptides.

## Figures and Tables

**Table 1:** Minimum inhibitory concentration (MIC) of polypeptide-enriched extracts from green leaves (protein content of extract 30 μg/mL) and rhizomes (protein content of extract 26 μg/mL) of *P. oceanica*. Values are expressed as MIC in % *v*/*v* or μg/mL of protein content in brackets.

	MIC	
	Green Leaves	Rhizomes
*S. aureus* ATCC 25923	25% *v*/*v* (7.5 μg/mL)	50% *v*/*v* (13 μg/mL)
*P. aeruginosa* ATCC 15442	12.5% *v*/*v* (3.75 μg/mL)	6.2% *v*/*v* (1.61 μg/mL)
*E. coli* ATCC 25922	25% *v*/*v* (7.5 μg/mL)	12.5% *v*/*v* (3.25 μg/mL)
*E. faecalis* ATCC 13813	25% *v*/*v* (7.5 μg/mL)	12.5% *v*/*v* (3.25 μg/mL)
*C. albicans* ATCC 10231	25% *v*/*v* (7.5 μg/mL)	12.5% *v*/*v* (3.25 μg/mL)

**Table 2:** Biofilm inhibition formation of polypeptide-enriched extracts of *P. oceanica*. Values are expressed in terms of BIC_50_ in μg/mL.

		BIC_50_		
	Green Leaves	Standard Deviation	Rhizomes	Standard Deviation
*S. aureus* ATCC 25923	0.67	±0.05	0.85	±0.08
*C. albicans* ATCC 10231	0.94	±0.08	0.36	±0.02

**Table 3:** Peptide sequences identified in *P. oceanica* extracts and their characteristics as AMPs.

*P. oceanica* Extract	#No.	Identified Sequence	Predicted Ability to Act as an AMP	Percentage of Similarity with Already-Studied AMPs
Green leaves	#1	MDSGGLLLR	-	44.44% TRP1-TINF Tachykinin related peptides I (insects)
	#2	EETFHGVL	Yes	38.46% PG-SPI (frogs, amphibians)
	#3	NVVELNVAPGDK	Yes	46.15% Acidocin LCHV (bacteria)
	#4	VNVEKVVAPAN	-	40% P4 (bacteria)
Rhizomes	#5	IVASVGSA	Yes	50% coB1 (bacteria)
	#6	LSVEVEQ	-	37.5% Fusaricidin B (bacteria)
	#7	GEFALCSAKT	Yes	40% EP5-1 (invertebrates)
	#8	VVSALPVVEPTST	-	46.15% Griselimycin (bacteria)
	#9	DCDDDCCCGDN	-	45.45% SAAP fraction 3 (mammals)

**Table 4:** Main physico-chemical parameters of selected peptides identified in polypeptide-enriched extracts from green leaves and rhizomes of *P. oceanica*.

	Peptide #2	Peptide #3	Peptide #5	Peptide #7
Peptide sequence	EETFHGVL	NVVELNVAPGDK	IVASVGSA	GEFALCSAKT
Monoisotopic theoretical mass (Da)	930.445	1253.662	702.391	1025.490
Net charge	−1.75	−1	0	0
Isoelectric point	5.385	5.723	5.909	5.878
Wimley–White whole-residue (kcal/mol)	2.74	5.36	0.44	1.7
Hydrophobic ratio (%)	38%	42%	63%	50%
Protein-binding potential Boman Index (kcal/mol)	0.99	1.21	−1.34	0.45
Half-life (s)	1.51	3.48	1.08	1.24
Stability in a biological proteolytic environment	High	High	High	High

**Table 5:** Predicted biological properties of selected peptides of *P. oceanica*.

Features	Peptide #2	Peptide #3	Peptide #5	Peptide #7
Sequence	EETFHGVL	NVVELNVAPGDK	IVASVGSA	GEFALCSAKT
CPP (cell-penetrating peptides) ^1^	No	No	No	No
Antibacterial activity (probability) ^2^	30.5%	5.7%	35%	43.1%
Antifungal activity (probability) ^2^	5.9%	4.4%	19%	48.8%
Antibiofilm activity ^3^	Yes	No	No	No
Hemolytic potential (probability) ^4^	0.49	0.49	0.49	0.48
Toxicity ^5^	No	No	No	No
Degradation by tripsin ^6^	No	Yes	No	Yes
Degradation by pepsin (pH = 1.3) ^6^	Yes	Yes	No	Yes
Degradation by pepsin (pH > 2) ^6^	Yes	Yes	No	Yes

^1^ CPP (cell-penetrating peptides): https://webs.iiitd.edu.in/raghava/cellppd/index.html, URL (accessed on 1 April 2022). ^2^ iAMPpred: http://cabgrid.res.in:8080/amppred/index.html, URL (accessed on 1 April 2022) ^3^ dPABBs: https://ab-openlab.csir.res.in/abp/antibiofilm/index.php, URL (accessed on 1 April 2022) ^4^ HemoPI: https://webs.iiitd.edu.in/raghava/hemopi/index.php, URL (accessed on 1 April 2022) ^5^ ToxinPred: https://webs.iiitd.edu.in/raghava/toxinpred/index.html, URL (accessed on 1 April 2022) ^6^ Peptide cutter server: https://web.expasy.org/peptide_cutter, URL (accessed on 1 April 2022).

**Table 6:** Some physico-chemical parameters of selected derivative synthetic peptides.

Natural Peptide Sequence	Derivative Synthetic Peptide	Derivative Synthetic Peptide Sequence	Monoisotopic Theoretical Mass (Da)	Net Charge	Boman Index (kcal/mol)	Hydrophobic Ratio (%)
EETFHGVL	Peptide# 2d	RKTFWGVL	1005.576	+2	0.97	50
NVVELNVAPGDK	Peptide# 3d	WVVRLNAPGKK	1266.756	+3	1.32	45
IVASVGSA	Peptide# 5d	IVAKVGSA	743.454	+1	−1.07	63
GEFALCSAKT	Peptide #7d	GEFALCKATK	1066.550	+1	0.67	50

**Table 7:** Predicted biological parameters of synthetic derivative peptides.

Synthetic Derivative Peptide	Antibiofilm Activity ^1^	CPP ^2^	Antimicrobial Activity ^3^	Antifungal Activity ^3^	Hemolytic Potential ^4^	Toxic Potential ^5^	Trypsin Cleavage Site ^6^	Pepsin Cleavage Site pH 1.3–pH > 2 ^6^
Peptide# 2d	Yes	No	45%	19%	0.51	No	Yes	Yes–Yes
Peptide# 3d	Yes	No	75%	31%	0.48	No	Yes	Yes–Yes
Peptide# 5d	Yes	No	87%	57%	0.49	No	Yes	No–No
Peptide# 7d	Yes	No	86%	87%	0.50	No	Yes	Yes–Yes

^1^ dPABBs: https://ab-openlab.csir.res.in/abp/antibiofilm/index.php, URL (accessed on 1 April 2022) ^2^ CPP (Cell-Penetrating Peptides): https://webs.iiitd.edu.in/raghava/cellppd/index.html, URL (accessed on 1 April 2022) ^3^ iAMPpred: http://cabgrid.res.in:8080/amppred/index.html, URL (accessed on 1 April 2022) ^4^ HemoPI: https://webs.iiitd.edu.in/raghava/hemopi/index.php, URL (accessed on 1 April 2022) ^5^ ToxinPred: https://webs.iiitd.edu.in/raghava/toxinpred/index.html, URL (accessed on 1 April 2022 ^6^ Peptide Cutter Server: https://web.expasy.org/peptide_cutter/, URL (accessed on 1 April 2022).

## Data Availability

The data presented in this study are available upon request from the corresponding author.
